# The Role of Symptom Duration and Serologic Factors in the Relapse of IgG4-Related Ophthalmic Disease following Surgery: A Retrospective Cohort Study

**DOI:** 10.1155/2022/5651506

**Published:** 2022-02-26

**Authors:** Siyu Liu, Zifan Yue, Chengcheng Zeng, Xiao Huang, Jian Li, Jiale Diao, Xinxin Chen, Ruili Wei, Weihua Yang

**Affiliations:** ^1^Department of Ophthalmology, Changzheng Hospital of Naval Medicine University, Shanghai, China 200003; ^2^Department of Ophthalmology, Naval Medical Center of PLA, Shanghai, China 200050; ^3^The Affiliated Eye Hospital of Nanjing Medical University, Nanjing, Jiangsu, China 210029; ^4^The Laboratory of Artificial Intelligence and Bigdata in Ophthalmology, 210029, China

## Abstract

IgG4-related disease (IgG4-RD) affects multiple organs and is characterized by immune-mediated inflammation and fibrosis; IgG-RD affecting orbital tissue is known as IgG4-related ophthalmic disease (IgG4-ROD). This research is aimed at exploring whether symptom duration and common serologic factors, such as IgG, IgE, and eosinophils, are potential risk factors for IgG4-ROD patient relapse after surgery and identifying possible causes of the positive correlation between symptom duration and relapse. This retrospective cohort study included 40 IgG4-ROD patients after surgery. Auxiliary inspection results were obtained before surgery and during follow-up, and relapse risk factors were identified based on previous studies. We used the Spearman rank correlation test to reveal the relationship between symptom duration and relapse time and identified the optimal cutoff value for symptom duration by X-tile. Then, we divided the patients into the long-duration and short-duration groups. Kaplan–Meier survival analyses and log-rank tests were performed to identify the relationship between symptom duration and relapse using X-tile software. Finally, we studied the relationship between previously studied relapse risk factors and symptom duration. The survival curves of the long-duration and short-duration groups were obviously different, and the baseline serum IgG, IgE, and eosinophil levels and asthma concomitant rate were significantly different between the long-duration and short-duration groups. Furthermore, the baseline serum IgG (*r* = 0.485, *P* = 0.002), IgE (*r* = 0.350, *P* = 0.037), and eosinophil (*r* = 0.6535, *P* < 0.0001) levels were positively correlated with symptom duration. Our study shows that IgG4-ROD symptom duration is significantly positively correlated with relapse rate and negatively correlated with relapse time. Symptom duration was positively correlated with serum baseline IgG4, IgE, and eosinophil levels and asthma history, which were potential risk factors for disease relapse. We recommended that IgG4-ROD patients with symptom durations greater than 96 months continue to receive maintenance steroid therapy longer than 1 year postsurgery to reduce the relapse rate.

## 1. Introduction

IgG4-related disease (IgG4-RD) is an immune-mediated inflammatory disorder with fibrosis that can affect multiple organs, and the affected organs can have tumor-like lesions and even failure [1]. The histopathological characteristics of IgG4-RD are abundant IgG4-positive plasma cell infiltration, numerous lymphocyte and plasma cell infiltration, phlebitis obliterans, and mat-pattern fibrosis [2], and the gold standard for the diagnosis of IgG4-RD is its characteristic histopathology accompanied by a significant infiltration of IgG4+ plasma cells [3]. IgG4-RD can affect multiple organs of the human body, including the lacrimal glands, salivary glands, aorta, bile ducts, pancreas, lungs, kidneys, liver, and dura mater. The clinical features of IgG4-RD include autoimmune pancreatitis, Mikulitz disease, retroperitoneal fibrosis, orbital inflammatory pseudotumor, autoimmune cholangitis, hypertrophic dura meningitis, and interstitial pneumonia [4]. IgG-RD that affects orbital tissue is also known as IgG4-related ophthalmic disease (IgG4-ROD).

More and more attention paid to the diagnosis and treatment of this infrequent autoimmune disease since the publication of diagnostic criteria for IgG4-RD [5]. In 2015, the first international consensus guidance statement which provided standardized management and treatment of IgG4-RD was published [6]. The statement called for all active IgG4-RD and IgG4-ROD patients to receive treatment, and the first-line drug for remission induction is glucocorticoids. For patients whose disease progression cannot be controlled by glucocorticoid monotherapy, a combination of glucocorticoid and immunosuppressant therapy is required, and B cell depletion therapy and surgery are also reasonable treatment options. However, each treatment method is accompanied by different degrees of relapse [7, 8], and the pattern of disease relapse is still unpredictable [9]. How to maintain treatment to avoid relapse is still an urgent problem to be solved, and a better understanding of risk factors for relapse plays an important role in disease management and relapse monitoring. The risk factors for relapse of IgG4-RD include an increase in serum IgE, mast cells, eosinophils, and a history of asthma [10, 11]. However, due to the heterogeneity and rarity of the disease, there are still few studies on the influencing factors of IgG-ROD relapse, especially in patients who have undergone surgical treatment.

Our study is a retrospective cohort study that included 40 IgG4-ROD patients after surgery. Our study describes the clinical characteristics and differences in serological indicators of each patient, explores and analyses whether symptom duration and serologic factors, such as serum IgE and IgG4, are potential risk factors for IgG4-ROD patient relapse after surgery, and tries to identify possible causes of the positive correlation between the duration of symptoms and relapse. We hope that this study will provide reliable predictors of relapse for IgG4-ROD and new recommendations for the rational treatment of IgG4-ROD.

## 2. Materials and Methods

### 2.1. Cohort Conduction and Patient Selection

This research was permitted by the Ethics Committee of Changzheng Hospital affiliated with Naval Military Medical University. All the patients enrolled in the study signed an informed consent form, and all of our procedures for experimental implementation complied with the Declaration of Helsinki. We included all IgG4-ROD patients who met the inclusion criteria treated in Changzheng Hospital from March 2013 to July 2020 in the cohort (*n* = 40), and 4 patients were excluded from our cohort due to loss of follow-up. We diagnosed 21 (58.3%), 4 (11.1%), and 11 (30.6%) patients with definite, probable, and possible IgG4-ROD, respectively, according to the 2015 IgG4-ROD diagnostic criteria [12]. The inclusion criteria were patients undergoing surgical treatment, and the postoperative follow-up time was more than 1 year. The patients included in the cohort had no history of other autoimmune diseases.

### 2.2. Auxiliary Inspection Results

Auxiliary inspections, which included serologic factor analysis, imaging examinations, other laboratory tests, and histological examinations, were conducted before surgery and during follow-up. The indices detected included complete blood cell counts, serum IgE levels, serum IgG and IgG subclass levels, serum eosinophil concentrations and ratios, and other necessary test results. All patients received imaging examinations, such as computed tomography (CT), ultrasound scanning (US), and magnetic resonance imaging (MRI). All tissues removed after surgery underwent pathological biopsy.

### 2.3. Assessing Duration of Symptoms and Disease Relapse

We determined the time of onset during detailed medical history inquiry and diagnosis and treatment records provided by the patient. The time of onset was defined as the time when positive symptoms or signs appeared or when clinically significant auxiliary examination results appeared. After the operation, the patients went to the ophthalmology clinic of Changzheng Hospital for related examinations and follow-up at 1 month and 6 months later. Afterward, we sent questionnaires to the patients to investigate the relapse situation every year. The relapse time is defined as the reappearance of positive symptoms and signs, as well as the reappearance of diagnostic laboratory tests and imaging findings. An increase in serum IgG4 concentration alone cannot be considered a disease relapse. We determined the time from onset to treatment as the duration of symptoms.

X-tile was used to identify the optimal cutoff value, and the statistical significance index was *P* < 0.05 [13]. The obtained cutoff value was used as the basis for grouping the long-duration group and the short-duration group. Kaplan–Meier survival analyses and log-rank tests were performed to identify the relationship between the duration of symptoms and relapse using X-tile software.

### 2.4. Confirmation of Potential Predictors of Disease Relapse

The predictors screened in this experiment to predict disease relapse include some common clinical features of IgG4-ROD, past medical history, and serological indicators, like gender ratio, the rates of having a history of asthma, baseline serum IgG4, IgE, IgG, and eosinophil counts, which have been suggested by previous studies to be predictors of IgG4-RD relapse [8, 10, 11, 14, 15].

### 2.5. Statistical Analysis

All patient clinical data were gathered and are listed in the tables and supplementary materials of this article. We present distributed normally distributed quantitative variables as the means ± SDs and present nonnormally distributed features as the medians (IQRs). We compared continuous variables using the Mann–Whitney test. The Spearman rank correlation test was used to quantify the correlations. We used the chi-square test to compare categorical variables. Then, we generated all figures with GraphPad Prism version 8.1.1 (GraphPad Software, Inc., La Jolla, CA, USA) and performed all statistical analyses with SPSS Statistics version 24.0 (IBM Corp., Armonk, NY, USA).

## 3. Results

### 3.1. Baseline Features of the Patients Enrolled in This Cohort

We enrolled 40 patients in our cohort. After excluding 4 patients who were missing the follow-up, 36 patients remained, and their clinical and demographic baseline features are shown in Table 1. The average age at onset was 52.86 years, and the sex ratio was 3.5, with male patients accounting for the majority of the cohort. In addition, the average follow-up time was 40.64 months. Notably, the average time from onset to treatment was 64.22 months, and the relapse rate after surgery was 38.9%.

According to the clinical characteristics, eyelid swelling (77.8%) and exophthalmos (52.8%) were the most common clinical symptoms in IgG4-ROD patients, and the lacrimal gland was the most commonly affected organ/anatomical site (88.9%). The proportion of asthma history was 36.1%.

### 3.2. Survival Analysis Construction and Cutoff Value Identification

**Figure 1:**
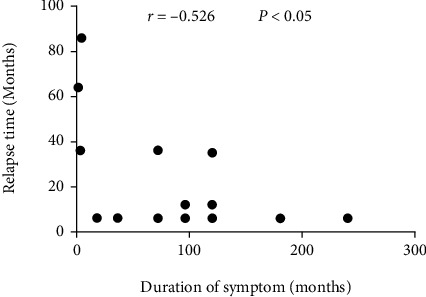
Comparison of the duration of symptoms and the relapse time by Spearman rank correlation tests. *P* values < 0.05 were considered significant.

**Figure 2:**
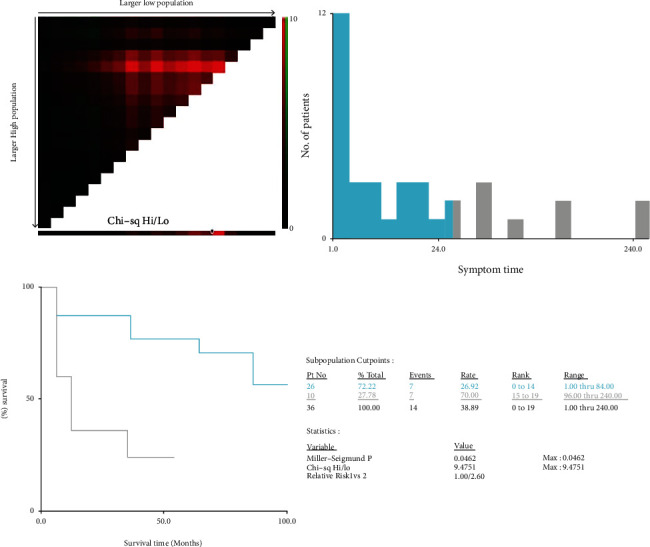
Identification of the cutoff value by X-tile software according to the duration of symptoms and the disease relapse time and rate. Each graph contains the X-tile plot, a histogram, the K-M curve, and the data related to the optimal cutoff point.

**Figure 3:**
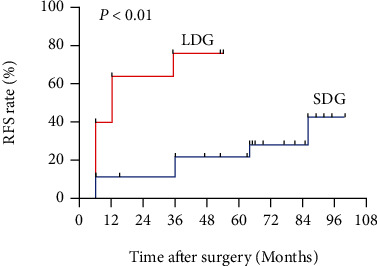
Survival curves of patients in the long-duration group (LDG) and the short-duration group (SDG). The relapse rate (RFS rate) of the long-duration group was higher than that of the short-duration group (*P* < 0.001).

The Spearman rank correlation test was used to reveal the relationship between the duration of symptoms and the relapse time, and the results are presented in Figure 1. The Spearman rank correlation test shows that there is a negative correlation between the duration of symptoms and the relapse time (*r* = −0.526, *P* < 0.05). We performed Kaplan–Meier survival analyses and log-rank tests to identify the potential relationship between the duration of symptoms and relapse. Based on the cutoff value calculated by X-tile (duration of symptoms = 96 months, the result is shown in Figure 2); we divided the 36 patients into a long-duration group (*n* = 10) and a short-duration group (*n* = 26). The survival curves of the IgG4-ROD patients in the long-duration group and short-duration group are shown in Figure 3. The log-rank test results revealed that the survival curves of the long-duration group and short-duration group were significantly different (*P* < 0.05), and the relative risk (RR) was 2.6 (95% CI 1.225-5.516), which indicates that symptom duration is strongly correlated with the disease relapse rate.

### 3.3. Differences in Clinical Features between the Long-Duration Group and the Short-Duration Group

A comparison of the baseline clinical characteristics between the patients in the two groups is presented in Table 2. According to the results, we found that the probability of asthma history was higher in the long-duration group, which means that patients with a long duration of symptoms had a higher probability of having asthma. The difference in the relapse rate of IgG4-ROD between the patients in the long-duration group and short-duration group was also statistically significant, and the long-duration group had a higher relapse rate. There were no significant differences in other clinical indicators, including the rates of eyelid swelling, exophthalmia, vision decrease, eyelid hyperemia, and diplopia, between the patients in the long-duration group and short-duration group.

**Figure 4:**
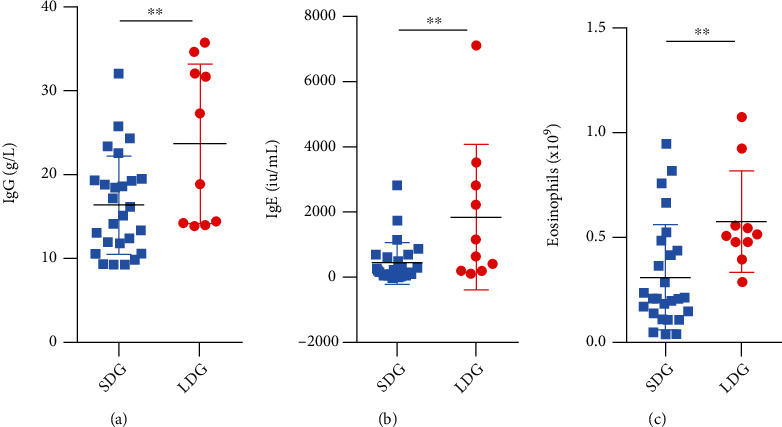
Comparison of baseline serum IgG, IgE, and eosinophil levels between patients in the long-duration group and the short-duration group. The horizontal bars represent the standard errors of the means. *P* values < 0.05 were considered significant. (a) Patients in the long-duration group and short-duration group presenting with elevated serum IgG levels. (b) Patients in the long-duration group and short-duration group presenting with elevated serum IgE levels. (c) Patients in the long-duration group and short-duration group presenting with elevated serum eosinophils levels.

There are differences in serological indicators between the long-duration group and the short-duration group. Mann–Whitney tests were performed to analyze whether there were significant differences in serological indicators, including serum IgG4, IgG, IgE, eosinophil, and globulin levels, between the long-duration group and short-duration group. The results are presented in Table 3 and Figure 4.

According to the results, we found that the levels of serum IgE, IgG, and eosinophils were significantly different between the long-duration group and short-duration group, which means that patients with a long duration of symptoms have higher baseline serum IgE, IgG, and eosinophil levels.

### 3.4. Identification of the Relationship between Risk Factors for Relapse and Duration of Symptoms

**Figure 5:**
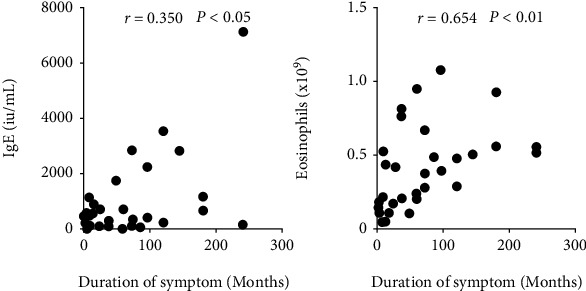
Comparison of eosinophil counts and serum IgE levels by Spearman rank correlation tests. *P* values < 0.05 were considered significant.

We used the Spearman rank correlation test to identify the relationships between baseline serum IgE and eosinophil levels and symptom duration, and chi-square tests were conducted to verify the relationship between the duration of symptoms and the history of associated asthma. The results are presented in Figure 5. Specifically, the results showed that serum IgE (*r* = 0.350, *P* = 0.037) and eosinophil (*r* = 0.654, *P* < 0.0001) levels were positively correlated with the duration of symptoms, and the chi-square test results between the long-duration group and short-duration group were also significantly different. This suggests a longer symptom duration along with higher serum IgE and eosinophil levels, and patients with a longer symptom duration are more likely to suffer from asthma.

## 4. Discussion

IgG4-related ophthalmic disease is a fibroinflammatory disease mostly characterized by IgG4^+^ plasma cell invasion. The lacrimal gland is the most common anatomical site of IgG4-ROD involvement [16, 17], and eyelid swelling, exophthalmos, and diplopia were the most common clinical symptoms [18]; our research results were consistent with these previous findings. In addition, IgG4-ROD can also affect eyelids, extraocular muscles, orbital adipose tissue, the optic nerve, conjunctiva, the trigeminal nerve, orbital bone, the lacrimal sac, sclera, choroid, and other ocular accessory tissues and adjacent orbital tissues [19, 20] and can cause symptoms, such as diplopia, orbital pain, restrictive eye movement disorder, decreased vision, tearing, and conjunctival congestion [18].

IgG4-ROD is a chronic illness, and the development of this disease involves a process of remission-relapse-remission [21]. How to effectively reduce the relapse rate has become a major problem in the treatment of IgG4-related ophthalmic disease, and identification of the risk factors could improve the management and probably decrease the relapse rate of IgG4-ROD. Regarding therapeutic methods, glucocorticoids are the first-line treatment for most patients with IgG4-ROD. In particular, systemic glucocorticoid therapy should be the first choice for patients with active IgG4-ROD [19]. However, glucocorticoid therapy has a high relapse rate. Ebbo et al. [22] found that 68.4% of IgG4-ROD patients relapsed after a first course of glucocorticoids in their cohort, and Karim found that including IgG4-ROD, approximately 62% of IgG4-RD patients relapsed in their cohort [23]. Furthermore, glucocorticoid therapy does not respond particularly well to many hormone-intolerant patients and elderly people, and the cycle of glucocorticoid therapy is also relatively long. Tacelli et al. revealed that maintenance steroid therapy lasting longer than 1 year could reduce the risk of relapse [24]. Surgical treatment as an alternative treatment for IgG-ROD has been shown to have a lower relapse rate than glucocorticoid therapy [25]. All the IgG4-ROD patients in our cohort had a relapse rate of 38.9%, and in particular, those in the short-duration group had a relapse rate of 26.9%; these findings confirmed the results of previous studies, suggesting that surgical treatment for IgG4-ROD likely has a lower relapse rate than glucocorticoid treatment. This provides a new direction of rational treatment for IgG4-ROD patients, especially those with glucocorticoid intolerance and elderly people. In addition, researchers found that low-dose steroid maintenance therapy significantly reduced the relapse rate [1, 2]. However, long-term glucocorticoid treatment has many complications, such as Cushing's syndrome, osteoporosis, hypertension, and diabetes [26, 27]. If doctors can correctly identify patients at high risk of relapse and target them with low-dose steroid, maintenance therapy could lead to more rational treatment strategies for IgG4-ROD.

From our survival analysis results, we realized that there was a potential relationship between symptom duration and disease relapse: as the duration of symptoms increased, the relapse time was earlier, and the relapse rate also increased. Liu et al. [8] found that the duration from diagnosis to treatment was an independent risk factor for relapse in IgG4-RD patients. However, there is no previous independent study on the relationship between IgG4-ROD and the duration of symptoms. Our research proves that the duration of symptoms is a risk factor for disease relapse and provides theoretical support for the rationality of early diagnosis and treatment of IgG4-ROD.

To further explore the reasons for the high relapse rate of IgG-ROD due to the long duration of symptoms, we conducted a study on the relationships between common serological and clinical indicators and the duration of symptoms. The results show that a longer symptom duration causes higher serum IgE and eosinophil baseline levels, and patients with a longer symptom duration were more likely to develop asthma. Interestingly, serum baseline levels of IgE and eosinophils and history of asthma are all risk factors for IgG4-ROD relapse. Wallace et al. [15] found that baseline elevations in both serum IgE and blood eosinophil concentrations independently predict IgG4-RD, including IgG4-ROD relapses. Zhou et al. [11] found that IgG4-RD patients (including IgG4-ROD patients) with high serum IgE levels at baseline were more likely to have higher disease activity and higher disease relapse rates. Liu et al. [8] found that the risk factors for IgG4-ROD relapse required a longer duration from diagnosis to treatment and a history of allergy. Della Torre et al. [14] found that increased serum IgE and eosinophil levels are independent of atopic disease history, revealing that these factors reflect mechanisms inherent to the immune response driving IgG4-RD; they also found that T-helper type 2 (Th2) cells play crucial roles in IgG4-RD pathogenesis [14] and proved that the IgG4-RD Th2-polarized immune state is related to antecedent atopic disease rather than disease activity [28]. We speculated that the serum IgE and eosinophil levels might reflect the different immune statuses of the body. Patients with high serum indexes are more sensitive to the immune response, which makes the disease more likely to relapse. Furthermore, Th2 cells can secrete specific cytokines, such as IL-13, IL-5, and IL-4, to cause asthma and immune abnormalities [29], and the presence of asthma can increase the number of circulating Th2 memory cells, which increases atopy and enhances Th2 responses, such as increasing serum IgE and eosinophil levels, in IgG4-RD [28, 30]; this phenomenon explains why the relapse rate of patients with a history of asthma is increased. In addition, our research verified the positive correlation between serum baseline levels of IgG, IgE, eosinophils, history of asthma, and symptom duration in IgG4-ROD patients, indicating that as the duration of symptoms increases, serum baseline levels of IgE and eosinophils accumulate in the body and are more likely to accompany asthma. These factors induce IgG4-ROD relapse and suggest that patients with IgG4-ROD should undergo surgery as soon as possible to reduce the duration of symptoms to reduce the relapse rate. This is probably due to the increasing number of circulating Th2 memory cells with prolonged symptom duration because Th2 memory cells secrete a large number of cytokines, such as IL-4 and IL-13, which leads to the accumulation of serum IgE and eosinophils and the occurrence of asthma. However, whether the above high-concentration serological indicators and high asthma concomitant rate are the cause of disease relapse still needs follow-up prospective studies and animal experiments for verification.

## 5. Limitations

Our research was retrospective, selection bias and recall bias probably occurred, and measurement bias may exist in different patients due to the different testing times and personnel of serological indicators. Furthermore, the sample size of the research is small and does not conform to the normal distribution, so many reasonable statistical methods cannot be performed. Whether the duration of symptoms is indeed an independent risk factor for relapse still needs to be further explored by expanding the sample size and using Cox regression analysis and other more convincing statistical methods. Moreover, due to the scarcity of IgG4-ROD patients and the limitations of retrospective studies, the experiment did not adopt 1 : 1 grouping. The patient's disease relapse time may not be completely accurate due to the limitation of the follow-up period. The duration of symptoms completely depended on patient self-report; consequently, the possibility of memory bias existed, although we carefully verified symptom duration. We plan to further explore whether the duration of symptoms is indeed an independent risk factor for relapse by expanding the sample size and using more convincing statistical methods in our future research.

## 6. Conclusions

In conclusion, our research used the largest known cohort of patients undergoing surgical treatment for IgG4-ROD and for the first time explored the relationship between the duration of symptoms and the relapse of IgG4-ROD. Our study provides novel information about risk factors for IgG4-ROD relapse and found that the duration of symptoms of IgG4-ROD has a significant positive correlation with relapse of the disease. As the duration of symptoms increases, the relapse time becomes earlier, and the relapse rate also increases. The duration of symptoms is positively correlated with serum baseline levels of IgE, eosinophils, and asthma history, which are risk factors for disease relapse, suggesting that the prolonged duration of symptoms may lead to the accumulation of risk factors for the relapse of the abovementioned diseases and lead to relapse. For these reasons, we recommended that IgG4-ROD patients with symptom durations greater than 96 months continue to receive maintenance steroid therapy longer than 1 year after surgery to reduce the relapse rate. Our research provides theoretical support for advocating early treatment of IgG4-ROD and for the reasonable selection of treatment options; additionally, our study provides new directions and ideas for follow-up studies on the prevention of IgG4-ROD relapse and the identification of potential serologic biomarkers for predicting disease relapse.

## Figures and Tables

**Table 1:** Baseline clinical characteristics of patients with IgG4-related ophthalmic disease (*n* = 36).

Variables	Value
Age (mean ± SD), years	52.9 ± 16.4
Sex (male/female)	28/8
Laterality (unilateral/bilateral)	13/23
Duration of symptoms, median (IQR), months	42 (9-96)
IgG4-ROD diagnosis (definite/probable/possible)	21/4/11
Orbital lesion	
Lacrimal glands (%)	32 (88.9)
Extraocular muscles (%)	14 (38.9)
Orbital fat (%)	4 (11.1)
Optic nerve (%)	3 (8.3)
Trigeminal nerve (%)	9 (25.0)
Clinical symptoms	
Eyelid hyperemia (%)	13 (36.1)
Diplopia (%)	9 (25.0)
Eyelid swelling (%)	28 (77.8)
Exophthalmia (%)	19 (52.8)
Decreased vision (%)	4 (11.1)
Paranasal sinusitis (%)	28 (77.8)
Asthma (%)	13 (36.1)

**Table 2:** Baseline clinical characteristics of patients in the short- and long-duration groups.

Variables	Short symptom duration	Long symptom duration	*P* value	Variables	Short symptom duration	Long symptom duration	*P* value
Gender			0.397	Lacrimal glands			0.305
Male	19 (73.1%)	9 (90.0%)		Yes	24 (92.3%)	8 (80.0%)	
Female	7 (26.9%)	1 (10.0%)		No	2 (7.7%)	2 (20.0%)	
Laterality			0.270	Extraocular muscles			0.462
Unilateral	11 (42.3%)	2 (20.0%)		Yes	9 (34.6%)	5 (50.0%)	
Bilateral	15 (57.7%)	8 (80.0%)		No	17 (65.4%)	5 (50.0%)	
Smoking history			0.179	Orbital fat			0.057
Yes	4 (15.4%)	4 (40.0%)		Yes	1 (3.9%)	3 (30.0%)	
No	22 (84.6%)	6 (60.0%)		No	25 (96.2%)	7 (70.0%)	
Diplopia			0.686	Optic nerve			0.181
Yes	6 (23.1%)	3 (30.0%)		Yes	1 (3.9%)	2 (20.0%)	
No	20 (76.9%)	7 (70.0%)		No	25 (96.2%)	8 (80.0%)	
Eyelid hyperemia			1.000	Trigeminal nerve			0.226
Yes	9 (34.6%)	4 (40.0%)		Yes	5 (19.2%)	4 (40.0%)	
No	17 (65.4%)	6 (60.0%)		No	21 (80.8%)	6 (60.0%)	
Decreased vision			0.057	Paranasal sinusitis			0.397
Yes	1 (3.9%)	3 (30.0%)		Yes	19 (73.1%)	9 (90.0%)	
No	25 (96.2%)	7 (70.0%)		No	7 (26.9%)	1 (10.0%)	
Eyelid swelling			0.076	Asthma			**0.018**
Yes	18 (69.2%)	10 (100.0%)		Yes	6 (23.1%)	7 (70.0%)	
No	8 (30.8%)	0 (0.0%)		No	20 (76.9%)	3 (30.0%)	
Exophthalmia			0.274	Recurrence			**0.026**
Yes	12 (46.2%)	7 (70.0%)		Yes	7 (26.9%)	7 (70.0%)	
No	14 (53.9%)	3 (30.0%)		No	19 (73.1%)	3 (30.0%)	

^a^Continuous correction chi-square test. ^b^Fisher exact test.

**Table 3:** Baseline serological test in the short- and long-symptom-duration groups.

Variables	Short symptom duration	Long symptom duration	*P* value
Serum IgG (g/L)	16.42 ± 5.90	23.74 ± 9.45	0.030
Serum IgG4 (g/L)	9.27 ± 10.74	10.37 ± 7.68	0.417
Serum IgG4/IgG (%)	0.48 ± 0.36	0.46 ± 0.25	0.972
Serum IgM (g/L)	0.98 ± 0.42	1.01 ± 0.76	0.536
Serum IgE (IU/mL)	457.22 ± 636.89	1864.70 ± 2210.19	0.011
Serum IgA (g/L)	1.79 ± 0.71	1.68 ± 0.71	0.724
Serum eosinophilia (×10^9^/L)	0.31 ± 0.25	0.58 ± 0.24	0.004

Normal ranges: IgG 7-16 g/L; IgG4 <1.35 g/L; IgM 0.5-2.5 g/L; IgE <165 IU/mL; IgA 0.85-3 g/L; eosinophilia 0.02-0.52 (×10^9^/L).

## Data Availability

All of our research data can be found in the supplementary data we uploaded.
